# Early childhood intervention for children at risk of developmental disabilities and their caregivers in Rwanda: study protocol for the PDC/Baby Ubuntu cluster randomised trial

**DOI:** 10.1186/s13063-026-09613-7

**Published:** 2026-03-20

**Authors:** Nathaniel Scherer, Mathieu Nemerimana, Carol Nanyunja, Gatera Fiston Kitema, Samantha Sadoo, Fabrice Iradukunda, Mariella Munyuzangabo, Rachel Lassman, Sara Rotenberg, Irene Bagahirwa, Katie Greenland, Shanquan Chen, Mark Carew, Calum Davey, Giulia Greco, Lena Morgon Banks, David John Musendo, Francois Uwinkindi, Hannah Kuper, Emily L. Webb, Erick Baganizi, Cally J. Tann

**Affiliations:** 1 London School of Hygiene & Tropical Medicine, London, UK; 2 Partners In Health/Inshuti Mu Buzima, Kigali, Rwanda; 3 Medical Research Council/Uganda Virus Research Institute and London School of Hygiene & Tropical Medicine Uganda Research Unit, Entebbe, Uganda; 4 Lifetime Consulting and Partners, Kigali, Rwanda; 5 Rwanda Biomedical Centre, Kigali, Rwanda; 6 University College London Hospitals NHS Trust, London, UK

**Keywords:** Disability, Child, Caregiver, Participation, Quality of life, Randomised controlled trial, Process evaluation

## Abstract

**Background:**

Early childhood intervention strategies have the potential to promote health, participation and quality of life for young children at risk of developmental disabilities and their caregivers, however evidence on the impact of integrated care strategies in sub-Saharan Africa is lacking. Access to early intervention is crucial for affected children and families, particularly in resource-constrained settings with limited access to specialised services. This trial aims to evaluate the effectiveness and implementation of a bundle of early identification, care and support, integrated into government health systems in Rwanda: the Pediatric Development Clinic (PDC)/Baby Ubuntu programme.

**Methods:**

The study is a single-blind, effectiveness implementation-hybrid (type II) cluster randomised controlled trial with two arms (1:1 ratio). At cluster level, all community health centres in the three trial districts will be eligible for inclusion. At the participant level, at risk children aged ≤ 59 months will be eligible where ‘at risk’ is defined as being a survivor of a newborn condition that is a recognised risk factor for developmental disability (neonatal encephalopathy, prematurity, meningitis, severe jaundice, cerebral malaria, suspected genetic and chromosomal conditions and seizures), and/or not meeting age-specific developmental milestones. Those receiving inpatient hospital treatment or in institutional care will not be eligible. Primary outcomes will be family health-related quality of life (PedsQL) and child participation (Young Child Participation & Environment Measure) assessed 12 months after enrolment and randomisation. Secondary outcomes include caregiver knowledge and confidence (scored structured assessments), psychological distress (Self-Report Questionnaire), experience of disability-affiliated stigma (Affiliate Stigma Scale), and economic activity (time-use survey), in addition to child mortality, illness and hospitalisation, child development/function (Global Scales of Early Development, Malawi Developmental Assessment Tool, PEDI-CAT), and nutritional status (weight-for-age, height-for-age). Analysis will be by intention-to-treat, consisting of all randomised subjects analysed according to assigned study arm. Cluster-level analyses will assess intervention effect.

**Discussion:**

The trial utilises best practice methodology and frameworks to conduct rigorous and comprehensive impact, process and economic evaluation of the intervention implemented and is guided by a multi-disciplinary team and steering committee.

**Trial registration:**

ISRCTN, ISRCTN17523514. Retrospectively registered 24 July 2024, https://doi.org/10.1186/ISRCTN17523514

**Supplementary Information:**

The online version contains supplementary material available at 10.1186/s13063-026-09613-7.

## Background

Globally, more than 315 million children and young people are affected by health conditions that increase their risk of developmental disabilities [1]. In response, international policy increasingly advocates for attention on early child development and disability, especially in the first thousand days, a critical period for child development. Sustainable Development Goal target 4.2 aims to ensure all children have access to quality early childhood development and care, inclusive of young children with developmental disabilities [2]. Further, the *Nurturing Care Framework* provides a roadmap to meet the developmental needs of all children, although targeted and tiered support for those at risk of disabilities is clearly warranted [3]. Access to early intervention strategies for affected children and caregivers is crucial, however the majority live in low- and middle-income countries (LMICs) where access to specialised care may be limited [4]. Caregivers of children with developmental disabilities frequently experience stigmatisation, prejudice, institutionalisation and barriers to participation, that affect social, economic and health outcomes [1]. Whilst evidence-based interventions across the life course exist, there remains a critical evidence gap for effective, agile and integrated early intervention strategies for implementation in diverse global contexts.

Many common newborn conditions are associated with an increased risk of developmental disabilities. Whilst significant gains have been achieved in reducing neonatal mortality reduction worldwide [5], there has been less focus on longer-term morbidity and disability outcomes from conditions such as neonatal encephalopathy, prematurity and severe jaundice [6]. Delayed identification of developmental impairment and delay amongst high-risk infants can worsen long-term outcomes [7] and evidence, largely from high-income countries, suggests that care strategies that include early identification and intervention for at risk children may improve long-term child, caregiver and family outcomes [8]. Participatory approaches to promote caregiver knowledge and confidence can improve child participation and inclusion, both at home and in the community [9], and can support caregivers and families who experience stigma and discrimination, isolation and rising economic costs of caring [10, 11]. Early intervention programmes can promote access to care, create an improved caregiving environment and improve family quality of life [8, 9, 12], however, there is a paucity of data from LMICs [9, 13]. In addition, programmes are typically developed and implemented locally by non-governmental organisations, and almost all are targeted at older children (> 2 years) [9].

This cluster randomised controlled trial will evaluate the effectiveness and implementation of the Pediatric Development Clinic (PDC)/Baby Ubuntu programme, a bundle of early identification, care and support for young children (<5 years) at risk of developmental disabilities and their families, integrated with government health systems in Rwanda. The PDC provides targeted developmental monitoring and support for children at risk of developmental delay and disability due to perinatal risk factors, while Baby Ubuntu is a community-based, participatory, group programme of early care and expert parent peer support for children with developmental disabilities and their caregivers. The combined PDC/Baby Ubuntu programme aims to maximise caregiver and child quality of life and participation.

### Hypotheses, Aim and Objectives

#### Hypotheses

1. Primary effectiveness hypothesis: The PDC/Baby Ubuntu programme of early care and support for children at risk of developmental disabilities integrated into government health services in Rwanda is effective in improving family health-related quality of life and child participation, when compared to enhanced usual care.
2. Primary implementation hypothesis: Integration of the PDC/Baby Ubuntu programme into government health systems at district level in Rwanda is feasible, acceptable and results in high-fidelity adoption, promoting reach of tier one services for children with developmental disabilities and their families.

#### Aim

This research aims to evaluate the effectiveness and implementation (feasibility, acceptability, cost, sustainability) of the PDC/Baby Ubuntu programme of early care and support for young children at risk of developmental disabilities and their caregivers integrated into government health systems in Rwanda.

#### Objectives

Specific objectives of the study are to:

1. Evaluate the impact of the PDC/Baby Ubuntu programme on caregiver, family and child outcomes, including family health-related quality of life and child participation.
2. Evaluate the feasibility and acceptability of PDC/Baby Ubuntu when integrated into government health systems at district level.
3. Explore barriers and facilitators to sustainable implementation of the programme with fidelity when integrated into government health systems.
4. Evaluate the cost and cost-effectiveness of the integrated programme.

## Methods

This protocol has been developed in line with SPIRIT (Standard Protocol Items: Recommendations for Interventional Trials) recommendations [14]. The completed SPIRIT checklist can be found in Supplementary Material 1. The trial was retrospectively registered on the ISRCTN registry (10.1186/ISRCTN17523514) prior to completion of participant enrolment. Retrospective registration occurred as a result of changes to Rwanda Law (No. 058/2021) that occurred during the trial inception period, which meant that approval from the Rwandan National Cyber Security Authority was required before details of the trial could be publicly shared.

The primary hypotheses will be tested through a single-blind, effectiveness implementation-hybrid (type II) cluster randomised controlled trial (cRCT) with two arms (1:1 ratio). Intervention clusters will receive the PDC/Baby Ubuntu programme, and control clusters enhanced usual care. For the main trial, trial outcome measures will be assessed in all clusters at two time points; baseline (T0) and endline, 12 months after enrolment and randomisation of the trial clusters (T1). Embedded process and economic evaluations will strengthen our understanding of programme implementation and scale-up. We will use the RE-AIM framework to guide the planning and evaluation of the programme according to five key outcomes: Reach, Effectiveness, Adoption, Implementation and Maintenance [15].

### Study setting

Rwanda ranks 160 out of 188 countries in the United Nations Development Program Human Development Index [16]. Child mortality has declined rapidly in Rwanda over the last few decades, with increased government investment in healthcare and expanding neonatal care facilities [17]. However, approximately 10% of newborns are born prematurely [18] and 24% of children are not achieving age-expected developmental milestones [17].

Rwanda operates a decentralised, tiered public health system, from primary care to national referral hospitals. At primary level, health centres deliver preventative, curative and promotional services. Health centres coordinate community health workers, who provide targeted support in local communities and connect households to primary healthcare. At secondary level, district hospitals provide diagnosis, treatment and inpatient care for patients referred from primary health centres. District hospitals coordinate primary health centres and organise district resources, logistics and health campaigns. Finally, at tertiary level, national referral hospitals deliver specialised care not available at district level. Standard care for all young children in Rwanda (including those at risk of developmental disabilities) includes home visits by community healthcare workers until age two, standard primary healthcare services at health centres and hospitals, child immunization, and referral to rehabilitation services at secondary and tertiary hospitals. There is no standard national package of care specifically for children at risk of developmental disabilities.

**Fig. 1:**
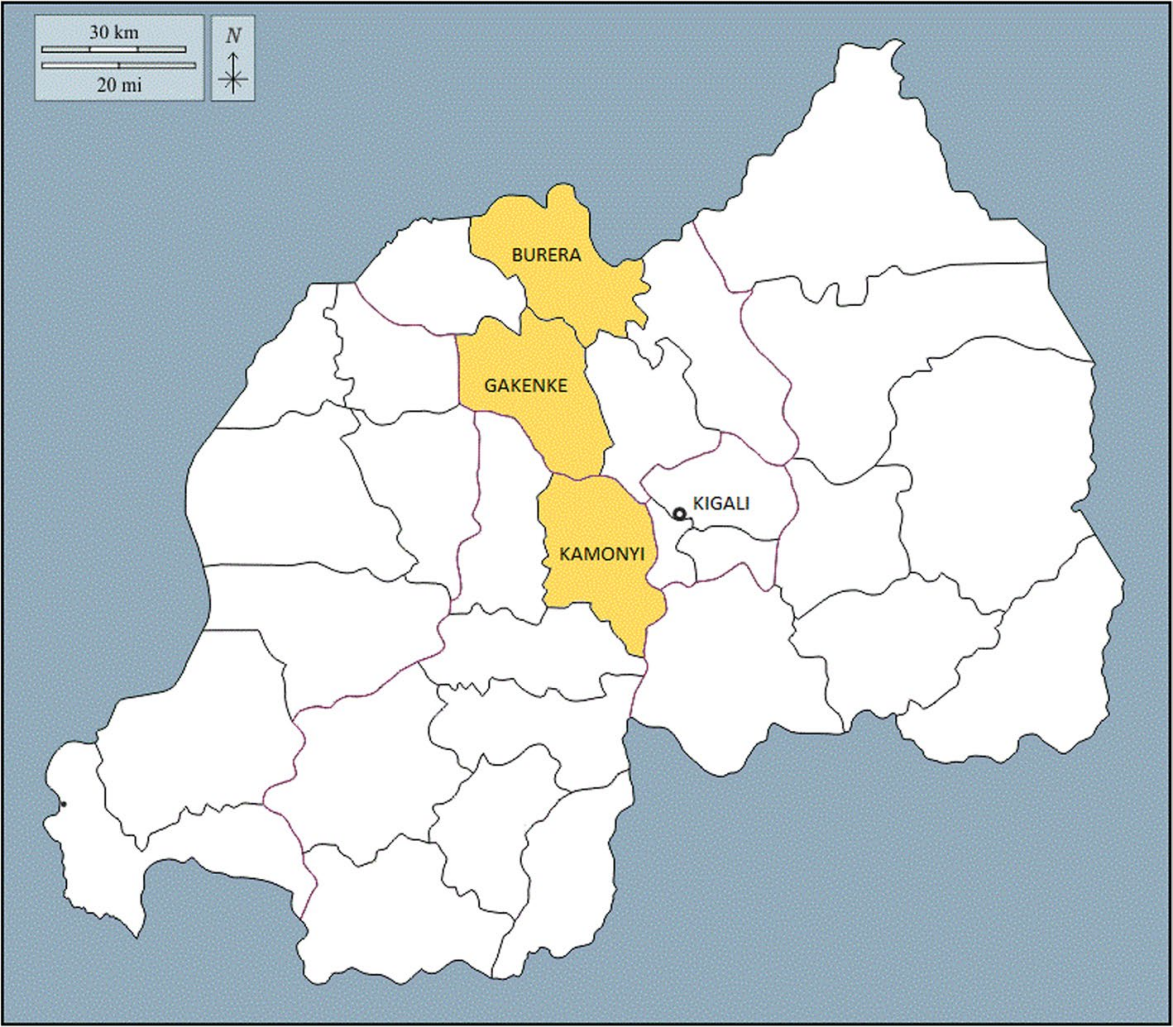
Map of Rwanda and Burera, Gakenke and Kamonyi districts

Three District Health Community Areas will be included in this trial: Burera, Gakenke, Kamonyi (Fig. 1). Burera is served by 19 health centres, Gakenke 23 health centres and Kamonyi 14 health centres. The unit of randomisation, a ‘cluster’, will be defined as the catchment area of two to three health centres in close geographic proximity. Each cluster will be surrounded by a buffer zone to limit contamination. In total, 20 clusters will be defined across the three districts; six clusters each in Kamonyi and Burera, eight clusters in Gakenke. In Supplementary Material 2 we have listed the health centres included in the trial and the total populations they serve.

### Study participants

Eligibility criteria:

Eligible participants will be children at risk of developmental disabilities aged 0–5 years and their caregivers, where ‘at risk’ is defined as children with:

- History of a newborn condition that is a recognised risk factor for developmental disability: prematurity, neonatal infection, neonatal encephalopathy (‘birth asphyxia’), severe jaundice, cerebral malaria, suspected genetic and chromosomal conditions, and seizures [1, 19–21].
- Not meeting age-specific developmental milestones, identified through routine child health service contact points, such as immunisation clinics and health screening by community health workers.
- Positive screening for developmental disabilities on an adapted version of the Ten Question Questionnaire, a brief screening tool for childhood disability [22].

Inclusion criteria:

- Children (0–59 months) meeting the definition of ‘at risk’ and their caregivers.
- Living within the catchment area of one of the health centres in a study cluster.
- Caregiver is willing and able to give written informed consent to take part in the study.
- Caregiver is either 18 years of age or older or, if younger than 18 years and the biological parent of the child, they are considered emancipated youth and eligible for participation.

Children requiring hospital treatment and/or living in institutional care at the time of the baseline assessment will not be eligible.

### Screening and recruitment

Prospective eligible participants (at risk children) in the three districts will be identified at the start of the project through an established network of government community healthcare workers and electronic medical records. Prospective participants will be invited to their local health centre for screening. After verbal assent from the child’s caregiver, trained data collectors will administer a simple screening questionnaire to confirm that the child meets the inclusion criteria. Should the child be eligible, data collectors will invite the child’s primary caregiver (defined as the caregiver who spends the most time with the child) to participate in the study, taking written informed parental consent. Enrolment will be offered to caregivers of all eligible children. Enrolment will continue until the required sample size is reached in each district. Children will be enrolled at their local health centre (defined as the health centre they visit for care) as opposed to the health centre to which they are registered, which may be geographically further and not where they practically access care.

### Randomisation

After baseline assessment, the 20 clusters will be randomised using a 1:1 allocation ratio to either the intervention arm receiving the PDC/Baby Ubuntu programme or the control arm receiving enhanced usual care. Randomisation will be stratified by district. Restricted randomisation will be used to ensure balance on cluster size which was determined to be potentially prognostic of intervention outcomes. The characteristics included in the restriction (district, cluster size) will be adjusted for in the analysis. Allocation will be completed by the trial statistician, not otherwise involved in the conduct of the trial. The outcome assessment team will be blinded to the allocation status of trial participants.

### Participant timeline

Table 1 outlines the schedule of enrolment, interventions and assessments. Primary and secondary outcomes will be assessed at two timepoints: baseline (T0), and 12 months later after randomisation (T1).

**Table 1:** Schedule of enrolment, assessment and intervention delivery

	Trial Period				
	Enrolment		Post-randomisation		Close-out
**Timepoint**	T0			T1	
**Enrolment**					
Eligibility screen	X				
Informed consent	X			X	
Baseline assessment	X				
Allocation		X			
**Intervention**					
Intervention arm			X	X	X
Control arm					X
**Assessment**					
Sociodemographic data	X				
Clinical characteristics	X				
Family health-related quality of life (PedsQL)	X			X	
Child participation (YC-PEM)	X			X	
Caregiver knowledge & confidence	X			X	
Caregiver psychological distress (SRQ)	X			X	
Caregiver experience of stigma (Affiliate Stigma Scale)	X			X	
Caregiver economic activity (time-use survey)	X			X	
Child survival (vital status)				X	
Child development (MDAT/GSED)	X			X	
Child function (PEDI-CAT)	X			X	
Child health (hospitalisations)				X	
Child anthropometric measurement (weight, height, OFC)	X			X	
**Process & economic evaluation**					
Qualitative data collection			X		
Economic data collection			X		

T0 = Baseline data collection; T1 = Endline data collection; YC-PEM = Young Children’s Participation and Environment Measure; PedsQL = Pediatric Quality of Life Inventory; MDAT = Malawi Development Assessment Tool; GSED = Global Scales for Early Development; PEDI-CAT = Pediatric Evaluation of Disability Inventory—Computer Adaptive Test; OFC = Occipital Frontal Circumference; SRQ = Self-Reporting Questionnaire

### Intervention description

The Pediatric Development Clinic (PDC) is an interdisciplinary model established in 2014 by Partners in Health/Inshuti mu Buzima (PIH/IMB) and the Rwanda Biomedical Centre (Ministry of Health) to monitor and provide targeted support within the public health system for children at risk of developmental delay and disability due to perinatal risk factors [23, 24]. Providers are non-specialised healthcare professionals, primarily general nurses and social workers. They are trained and mentored to deliver a structured package of neonatal follow-up and early intervention services based on components of nurturing care. Children enrolled in PDC are systematically followed up and are discharged from the programme at 36 months if they are developmentally on-track without any health or nutritional problems. PDCs implemented in Rwanda have demonstrated feasibility and improved child and caregiver outcomes [23, 24]. In a quasi-experimental study, post-neonatal unit mortality declined from 12 to 9% (p < 0.05), with a larger decline amongst preterm and low birth weight infants, reducing from 12.4% to 5.9% (p < 0.001).

Children attending the PDC who have severe developmental delay, assessed via the Guide for Monitoring Child Development (GMCD) [25], are referred to the Baby Ubuntu programme. Baby Ubuntu is a participatory, group-based, peer-led rehabilitation programme providing early care and support for young children with developmental disabilities and their caregivers. Group sessions are co-facilitated by community or facility-based healthcare professionals and ‘Expert Parents’, themselves parents of children with developmental disabilities. The content is divided into 11 modules, including information on understanding disability, positioning and carrying, feeding, communication, play, and experiences in the local community. Each session lasts for 2–3 hours. Evidence from mixed-methods, pre-post intervention evaluations of Baby Ubuntu in both urban and rural settings in Uganda have indicated a 20–25% increase in family and child health-related quality of life [26, 27]. A pilot feasibility, randomised-controlled trial in Uganda showed the programme to be both feasible and acceptable to families and health care workers in both rural and urban settings [28].

**Fig. 2:**
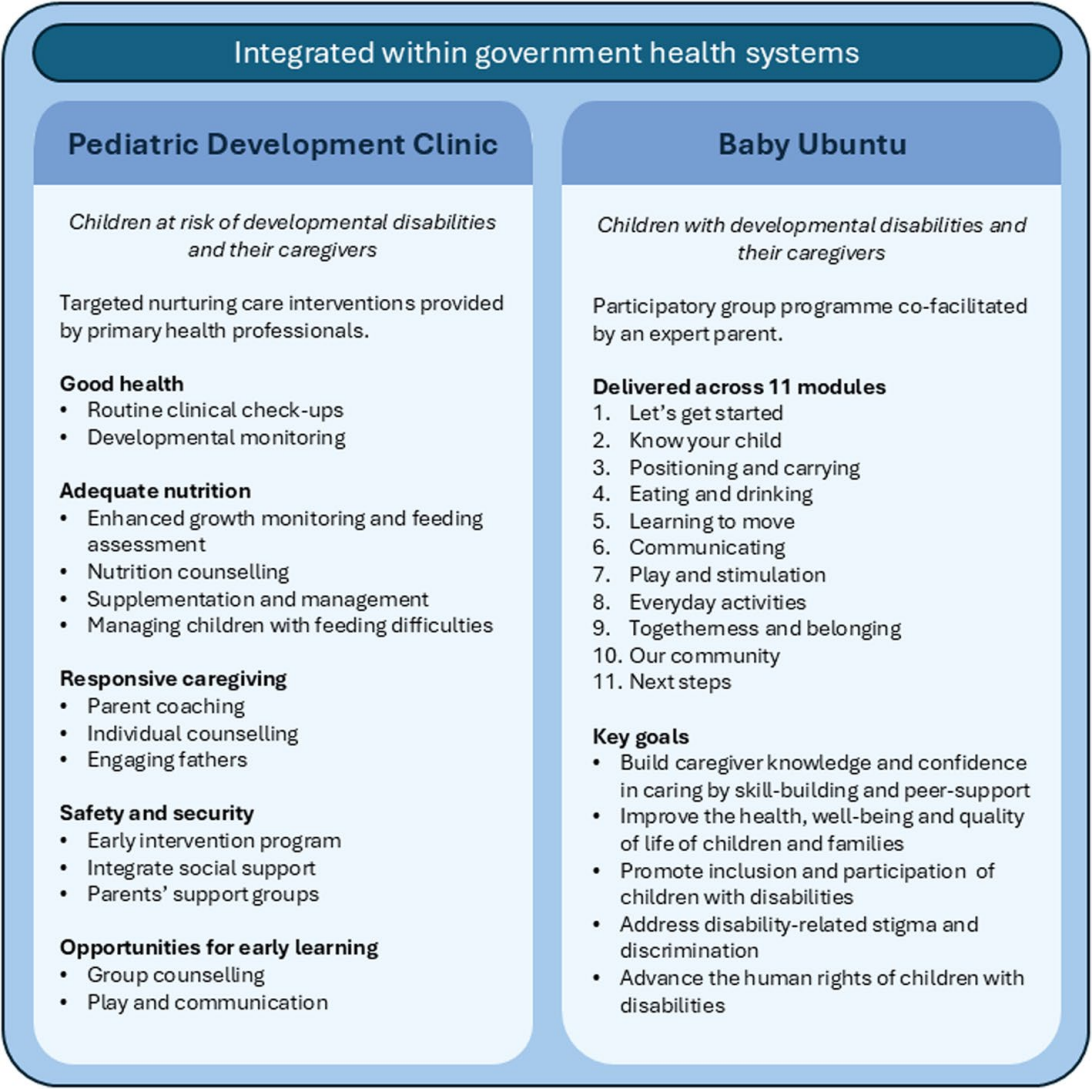
The Pediatric Development Clinic/Baby Ubuntu bundle

In 2019–20, the combined PDC/Baby Ubuntu bundle (Fig. 2) was piloted and evaluated in two rural districts of Rwanda integrated into government health systems [29]. Over three months, 10 PDCs enrolled 109 families to 12 Baby Ubuntu groups. Pre-post implementation evaluation showed a 20% increase in median family health-related quality of life score post intervention (62.5 vs. 79.9, p < 0.001). Significant improvements were seen in caregiver health-related quality of life and family functioning (p < 0.05). Satisfactory attendance was seen in 95% of families.

For this trial, the combined PDC/Baby Ubuntu bundle will be delivered in intervention cluster health centres as part of routine government child health services. Recruited children in the intervention clusters will be invited to the PDC at the health centre at which they completed the baseline survey. Children identified as having a developmental disability will be invited to join the Baby Ubuntu programme.

### Enhanced usual care

Specialised services for children at risk of developmental disabilities are uncommon in Rwanda. Families are frequently dependent on routine child health services from which they may be excluded. Evidence-based interventions for early care and support for at risk children are not available in most health centres.

Enhanced usual care will be provided to all participants, including those in the comparison arm. Participants will be given a printed handout listing available services, facility address and contact details. In the comparison arm, children will receive’enhanced usual care’ alone. There will be no restriction on concomitant care.

### Baseline data

Child and caregiver demographic information will be recorded at baseline through standardised questionnaires. Demographic information for the child will include date of birth, age and sex. Caregiver demographics data will include name, age, gender, marital status, address, contact details, relationship with child, education, employment status and religion. We will collect information on the head of household (if not the caregiver,) including age, education and employment status. Information on family characteristics will also be collected, including family size, ages of household members, family residence, household assets and household Ubudehe category, an income category assigned to households in Rwanda by the government. Principal Component Analysis will be used to determine participant socioeconomic status.

Disability status at baseline will be determined using the Malawi Developmental Assessment Tool (MDAT), a developmental screening tool developed and validated for use in Sub-Saharan Africa [30]. The tool assesses culturally appropriate age-standardised developmental milestones across four domains: gross motor, fine motor, language and social. Items are scored as pass (1) or fail (0). The child’s score in each domain is defined as the number of items completed until failure in six consecutive items. Development-for-age Z-scores (DAZ) are calculated in the MDAT Scoring Application in Shiny version 1.1 (https://kieran-bromley.shinyapps.io/mdat_scoring_shiny). Moderate to severe developmental disability will be defined as DAZ scores < −2 on endline assessment when children are older and diagnosis are more reliable.

### Effectiveness outcomes

Primary effectiveness outcomes are:

1. *Family health-related quality of life*: measured using the caregiver self-reported Pediatric Quality of Life Family Impact module (PedsQL) [31]. The PedsQL assesses the impact of pediatric chronic health conditions (here developmental disability and associated at risk conditions) on caregiver physical, social, emotional, cognitive, wellbeing, communication and worry, as well as the impact on family activity and family relationships. Scores are categorised into total, caregiver health-related quality of life and family functioning.
2. *Child participation*: measured using the Young Children’s Participation and Environment Measure (YC-PEM) [32]. The YC-PEM is a caregiver-completed measure that investigates the participation of children aged 0–5 in activities across home and the community. Scores are categorised into frequency, involvement and desire for change. The tool will be adapted and validated for use in this Rwandan population and setting.

Secondary effectiveness outcomes are:

- *Caregiver knowledge and confidence*: measured using an adapted version of the World Health Organization pre- and post-Parent Skills Training Test for Caregivers [33].
- *Caregiver psychological distress*: assessed via the Self-Reporting Questionnaire (SRQ) [34].
- *Caregiver experience of disability-related stigma*: measured using the Affiliate Stigma Scale [35].
- *Caregiver economic activity*: measured using a time-use survey from the Living Standards Measurement Survey [36].
- *Child mortality*: confirmed by caregiver report, community health worker or electronic medical records. Recorded at endline only.
- *Childhood hospital admission due to illness*: hospitalisations (defined as admission of one or more nights) in the past 12 months on caregiver report. Recorded at endline only.
- *Child development and function*: measured via the development-for-age Z-scores (DAZ) on the Global Scales of Early Development (age 0–3 years) [37], the Malawi Development Assessment Tool (age > 3 years) [30], and the Pediatric Evaluation of Disability Inventory - Computer Adaptive Test (PEDI-CAT) [38].
- *Child growth and nutritional status*: defined as weight-for-age (underweight), height-for-age (stunting), based on World Health Organization Growth Standards [39].

In addition, we will assess several exploratory outcomes. Experiences of early intervention will be assessed using the Family Outcomes Survey [40], child supervision and child discipline will be assessed using questions from UNICEF’s Multiple Indicator Cluster Survey (MICS) Questionnaire for children under five [41].The need for, and access to, disability-related goods and services, such as healthcare and assistive technology, will be recorded through caregiver report.

### Implementation outcomes

Primary implementation outcomes are feasibility and acceptability. Feasibility for families will be assessed by the proportion of those eligible to access the intervention who go on to utilise intervention services (attendance). Acceptability for families will be measured as the proportion with satisfactory programme adherence (≥ 60% sessions). Acceptability for community health centres will be defined as the proportion of approached health centres that go on to implement the programme. Secondary implementation outcomes will be fidelity, cost and cost-effectiveness (detailed under *Process and economic evaluation*).

### Data collection

Screening, informed consent and baseline/endline survey data collection will take place at health centres. Trained data collectors will collect survey data via structured interview with the child’s primary caregiver. Developmental progress will be assessed on direct assessment of the child using standardised, culturally-relevant developmental assessment tools. Data collectors will receive training for one week, including pilot for assessment and feedback. Pilot data will be used in the final analysis, dependent on data quality. All data collection tools will be translated into Kinyarwanda and back translated into English before finalisation. Data collection general procedures will be guided by a Standard Operating Protocol. Participants will be provided remuneration for participating in the baseline/endline survey and for transport (total 6,000 RWF).

### Blinding

The Principal Investigators and outcome assessment team will be blinded to the allocation status of trial participants. The outcome assessment team will be independent to the intervention delivery team and will be blinded to allocation at all outcome assessment timepoints. Given the nature of the intervention, it is not possible to blind participants. We do not anticipate any requirement for unblinding, but if required, the Principal Investigators will have access to group allocations and unblinding will be reported.

### Sample size

The primary analysis will be conducted amongst all children at risk of developmental disabilities and their caregivers. Secondary analysis will be conducted for the sub-population of children with developmental disabilities. Sample size calculations are based on the primary effectiveness outcomes amongst the sub-population of children with developmental disabilities, as this is the population for which data were available and is an important secondary analysis population. As a result, the trial will be powered to detect smaller intervention effects for the primary analysis population of all at risk children. We assume 10 clusters per trial arm with 35 children with developmental disabilities per cluster, for a total of 700 children with development disabilities overall, of whom 20% will not consent to participate in the evaluation or will be lost to follow-up. This results in a total of 560 children with developmental disabilities with outcome data (280 in each trial arm, 28 per cluster). Previously published data from a Ugandan cohort of children (< 3 years) with developmental disabilities suggest a mean family health-related quality of life score (PedsQL) of 60 amongst care as usual families (standard deviation 20) [28]. Data from Brazil suggest a mean YC-PEM home participation frequency raw score of 59 (standard deviation 7.5) and a mean YC-PEM home participation involvement raw score of 43 (standard deviation 7.5) in those receiving standard care [42]. Based on these values, Table 2 indicates detectable differences for family health-related quality of life and child participation among children with developmental disabilities, with 80% power and 5% significance level, for a range of intra-cluster correlation coefficient (ICC) scenarios. Detectable differences are shown as absolute difference, relative difference and as Cohen’s effect size.

**Table 2:** Detectable differences in family health-related quality of life and child participation primary outcomes

	**Intra-cluster correlation coefficient**		
Outcome	0.01	0.05	0.1
PedsQL^1^	Absolute difference: 5.6 Relative difference: 9.4%Cohen’s effect size: 0.28	Absolute difference: 7.7Relative difference: 12.8%Cohen’s effect size: 0.39	Absolute difference: 9.6Relative difference: 16.0%Cohen’s effect size: 0.49
YC-PEM frequency^2^	Absolute difference: 2.1Relative difference: 3.6%Cohen’s effect size: 0.28	Absolute difference: 2.9 Relative difference: 4.9%Cohen’s effect size: 0.38	Absolute difference: 3.6 Relative difference: 6.1%Cohen’s effect size: 0.48
YC-PEM involvement^3^	Absolute difference: 2.1Relative difference: 4.9%Cohen’s effect size: 0.28	Absolute difference: 2.9 Relative difference: 6.7%Cohen’s effect size: 0.38	Absolute difference: 3.6 Relative difference: 8.4%Cohen’s effect size: 0.48

^1^Assuming SD 20 and mean PedsQL score of 60 among control arm participants; ^2^Assuming SD 7.5 and mean YC-PEM frequency raw score of 59 among control arm participants; ^3^Assuming SD 7.5 and mean YC-PEM involvement score of 43 among control arm participants

Based on data from the pilot feasibility parallel arm randomised-controlled trial of Baby Ubuntu in Uganda [28], an intra-cluster correlation coefficient of 0.1 is a conservative assumption. We will therefore have 80% power to detect an absolute difference in mean PedsQL between trial arms of 9.6, and an absolute difference in mean YC-PEM frequency and involvement scores between trial arms of 3.6 and 3.6, respectively, equivalent to a Cohen’s effect size of 0.48–0.49. If the intra-cluster correlation coefficient is smaller, at 0.05, we will have 80% power to detect differences between trial arms of 7.7, 2.9, and 2.9 for these outcomes, respectively. These differences are smaller than those observed for the PedsQL in Uganda and the YC-PEM in Brazil [28, 42]. We estimate that half of children at risk of developmental disabilities will have a disability and will thus aim to recruit around 1,400 at risk children, inclusive of 700 at high risk of developmental disabilities.

### Data management

Trial data will be collected and stored in compliance with Rwanda Law No 058/2021 relating to the protection of personal data and privacy, the General Data Protection Regulation (GDPR) 2016/679 of the European Parliament and the Council of the European Union, and the London School of Hygiene & Tropical Medicine Research Data Management Policy. A Data Protection Impact Assessment will be developed and approved by the London School of Hygiene & Tropical Medicine Data Protection Officer to minimise data protection risks.

Hard copies of data (e.g. signed consent forms, qualitative interview notes) will be stored in locked, secure cabinets. Baseline and endline survey data will be collected electronically using REDCap on password protected tablets and uploaded to a secure, password-protected server in Rwanda. A master file linking participants’ names with ID numbers, signed consent forms, and contact information will be stored in an encrypted file separate from the data files.

Anonymised data will be shared with the London School of Hygiene & Tropical Medicine using a secure data-transfer protocol and stored on a secure, password-protected server. Twelve months after the end of the project, data will be made available on the London School of Hygiene & Tropical Medicine Data Compass (datacompass.lshtm.ac.uk), an open access public data repository, along with project documentation and a data-user guide. Data available on the repository will include no identifiers. Data will be made available upon request, provided the requester has a legitimate purpose for using the data. Participants will provide explicit consent for anonymised data to be made available on the Data Compass. Availability of data will be contingent on relevant approvals under Rwanda Law No 058/2021 relating to the protection of personal data and privacy.

### Data analysis

The trial will adopt a superiority design, evaluating whether the PDC/Baby Ubuntu intervention is superior to enhanced usual care in improving primary and secondary outcomes. Analyses will follow intention-to-treat principles. Clusters will be analysed according to their original randomisation, regardless of coverage or other variables.

The full analysis set will consist of all randomised subjects analysed according to the study arm to which they were assigned. The primary analysis will focus on all children at risk of developmental disabilities and their caregivers, comparing those receiving the PDC/Baby Ubuntu programme to the control group. The secondary analysis will compare children with developmental disabilities and their caregivers who received Baby Ubuntu to children with developmental disabilities in the control group. Superiority will be demonstrated if there are statistically significant improvements in the intervention arm versus the control arm for the prespecified outcomes.

Because of the relatively small number of clusters (n ≤ 20), cluster-level analyses will be used to assess the overall effect of the intervention. Specifically, in crude analyses, cluster-level means for each outcome measure will be generated and compared using t-tests. Adjustment for district and cluster size will be done using the two-stage approach described by Hayes and Moulton [43].

In further prespecified exploratory analyses, we will also analyse data at the individual level using generalised linear models (with an appropriate link function depending on the type of data) with random effects to allow for clustering of individuals. As this is not a confirmatory study, we do not plan any formal adjustment for multiple testing. Missing data will be handled using complete case analysis as the primary approach. Multiple imputation methods may also be used as a sensitivity analysis if substantial missing data exists (> 10% missing).

Subgroup analyses will assess whether the effect of the intervention may differ by variable, considering but not limited to socio-demographic status (e.g. maternal age, education, poverty), social support e.g. paternal abandonment/family structure/living situation; severity of disability; age of child at entry into the programme; locality (urban/rural). Any additional post-hoc analyses will be explicitly described as such in the final publication. Additional exploratory analyses will also consider non-randomised comparisons, such as investigation of dose response relationships within the intervention group.

### Process and economic evaluation

To investigate implementation outcomes and to inform scale-up, we will conduct a process evaluation complementary to the cRCT, guided by the RE-AIM framework across five key outcomes: Reach, Effectiveness, Adoption, Implementation and Maintenance [15]. The process evaluation will integrate quantitative and qualitative data. Quantitative outputs will include programme administration data, provider assessment and facility-level survey data. Qualitative methods will include in-depth interviews and focus group discussions with programme participants, providers and other key stakeholders.

Reach (the absolute number, proportion and representativeness of individuals who are willing to participate in the programme) will be measured through participant attendance records at the PDC and Baby Ubuntu. Qualitative methods will assess the barriers and facilitators to programme attendance. Feasibility and acceptability for families will be defined as previously stated. Effectiveness, assessed by the cRCT, will be complemented with qualitative data to evaluate perspectives of effectiveness and mechanisms of impact. Adoption (the absolute number, proportion and representativeness of settings willing to initiate the programme) will be measured by the proportion of health centres approached that implement the programme. Qualitative data will explore provider and stakeholders’ outlook on adoption. Implementation fidelity will be measured at PDC and Baby Ubuntu sessions through observation and structured fidelity checklist. Qualitative research will explore the barriers and facilitators to programme delivery. Maintenance (the extent to which a programme becomes part of routine practice) will be explored through qualitative research to consider perceptions and expectations of scale-up and institutionalisation of the PDC/Baby Ubuntu programme.

To determine the cost and cost-effectiveness of the programme, we will estimate the provider costs and an incremental cost-effectiveness ratio. Data will be collected from financial and administrative records, programme activity logs and discussions with providers (e.g. on time-use). In order to understand how costs may differ across settings, these data will be collected from purposively selected health centres to ensure variation by district, catchment area and participant number.

### Oversight and monitoring

The Trial Management Group (coordinating centre) is comprised of London School of Hygiene & Tropical Medicine, Partners in Health/Inshuti mu Buzima, the Rwanda Biomedical Centre, and Lifetime Consulting and Partners. The Trial Sponsor is the London School of Hygiene & Tropical Medicine (Keppel St, London WC1E 7HT). Neither the funder nor sponsor will have a role in study design, collection, management, analysis, interpretation of data, writing of the report and decision to publish.

The Trial Steering Committee (TSC) will provide overall supervision for the PDC/Baby Ubuntu trial on behalf of the Trial Sponsor and the Trial Funder to ensure that the trial is conducted according to the ICH guidelines for Good Clinical Practice, Research Governance Framework for Health and Social Care, and all relevant regulations and local policies. The TSC will have overall responsibility for the design and conduct of the trial and for safeguarding the rights, safety and well-being of participants. The TSC members will include the Principal Investigators, the Trial Statistician, the TSC Chair (an expert in child development/disability and evaluation trials), two independent experts in child development/disability and evaluation trials including a representative in Rwanda, and one representative from a disability organisation or caregiver association in Rwanda. The TSC will be guided by a TSC Charter, and membership is for the duration of the trial. If members leave the TSC, a replacement will be promptly appointed by the Chair. The TSC will meet online at regular intervals but not less than once every 12 months.

A Data Monitoring Committee (DMC) will be established independent of the investigators and the TSC. The DMC will safeguard the interests of the trial participants, assess the safety and efficacy of the intervention and monitor the conduct of the PDC/Baby Ubuntu trial. The DMC will report to the TSC and the Trial Sponsor. The DMC members will comprise the Chair (an expert on global child heath), a senior statistician, and a practitioner working in newborn and early child health research in Rwanda. The members will be independent of the Trial Management Group. The DMC will have access to all data on request. There will be no interim analysis or stopping criteria.

Internal monitoring will be conducted by the Trial Management Group for quality assurance and protocol adherence. The study is subject to audit by the London School of Hygiene & Tropical Medicine under their remit as Sponsor, as well as other regulatory bodies.

Any modifications to the protocol which may impact on the conduct of the study and patient safety, including changes of study objectives, study design, patient population, sample sizes, study procedures, or significant administrative aspects will require a formal amendment to the protocol. The amendment will be agreed by the Trial Management Group and approved by the ethics committees prior to implementation.

### Adverse event reporting

Adverse Events (AE) and Serious Adverse Events (SAE) will be recorded by the PDC/Baby Ubuntu Programme Coordinator and Trial Coordinator, with review and sign-off by the Principal Investigators. Adverse Events include any untoward or unfavourable medical or social occurrence in a research study participant, including any abnormal sign (e.g. abnormal physical exam or laboratory finding), symptom, disease, physical injury or social or emotional harm temporally associated with the participants’ involvement in the research, whether or not considered related to participation in the research. We define an SAE as any untoward medical occurrence that results in death, is life-threatening, requires hospitalisation, requires prolongation of existing hospitalisation, results in persistent or significant disability/incapacity, or requires intervention to prevent permanent impairment or damage. Unexpected and related SAEs are those that are not expected and considered to be related to the intervention. This definition captures the important events that are attributed to the intervention but do not follow known pattern of response.

Monitoring and reporting of AEs and SAEs will be guided by a Standard Operating Protocol. SAEs will be followed up by the Programme Coordinator, Trial Coordinator and Principal Investigators until their resolution or stabilisation or until causality is determined to be unrelated to trial interventions. Relatedness of an SAE to the programme will be determined by structured guidance. SAEs will be actively monitored in the control arm six months after the start of intervention implementation in order to ensure active monitoring and review of SAEs in both arms. An endpoint review committee, comprising two individuals experienced in paediatric care in Sub-Saharan Africa, will review all clinical information for children who die during the trial to determine the cause of death for use in the final analysis. The endpoint review committee will remain blinded to the randomised allocations.

The DMC will receive a summary of SAEs as requested. The DMC will note these and, unless there is a special cause of concern, will consider them as part of the planned DMC meetings which will discuss interim analyses. All SAEs will be reported to the Sponsor and ethical review committees.

### Patient and public involvement

The research questions, study design and outcome indicators were discussed in a full-day workshop in Rwanda with 48 stakeholders with experience and expertise in care and support for children at risk of developmental disabilities. Participants included caregivers of children with developmental disabilities, organisations of persons with disabilities (OPDs), non-governmental organisations, multilateral organisations, healthcare providers and policymakers. Study progress will be discussed with this stakeholder group at the midway point of the trial. Endline findings will be discussed with the group during data analysis to aid interpretation and prioritisation for dissemination.

### Dissemination

We will develop strategies for dissemination of findings to influence policy and practice, both in Rwanda and internationally. We will aim to publish trial results in reputable, peer-reviewed academic journals with open access. All manuscripts resulting from the trial will adhere to the CONSORT Guidelines, including the extension for cluster randomised trials [44]. We will also develop policy and evidence briefs. We will aim to present the study results at international meetings and conferences, such as the International Pediatric Association and Pediatric Academic Societies, and to key international audiences (e.g. World Health Organization, UNICEF) and funders.

## Discussion

Although early intervention has the potential to promote the quality of life and participation of young children at risk of developmental disabilities and their caregivers, there is limited evidence on early intervention strategies in sub-Saharan Africa. The PDC/Baby Ubuntu trial uses a cluster randomised controlled design to assess the effectiveness and implementation of a complex intervention on child and caregiver-relevant outcomes. Findings aim to inform strategies to integrate the PDC/Baby Ubuntu programme across government health systems in Rwanda. The study has several strengths. First, it applies evidence-based approaches and recognised evaluation frameworks to conduct rigorous impact, process and economic evaluation. Second, the methods and outcomes have been co-designed in participatory workshops with caregivers, persons with lived experience and experts in civil society, healthcare and government, to ensure relevance. Finally, the cluster randomised controlled trial design incorporates a large sample size, promoting statistical power and generalisability. In consideration of limitations, we note that whilst clusters have been created with sufficient geographic distance between them to limit contamination, this remains a risk and will be assessed at endline. Further, loss to follow-up for this largely rural at risk population may represent a challenge.

## Trial status

This manuscript is based on protocol version 2.4 (29 November 2024), approved by the Rwanda National Ethics Committee and the London School of Hygiene & Tropical Medicine Research Ethics Committee. Participant enrolment for the trial commenced on 3 March 2024 and recruitment ended on 2 November 2025. The trial is retrospectively registered on ISCRTN (ISRCTN17523514) as a result of delays in receiving necessary approvals that ensure adherence to Rwanda Law No 058/2021 relating to the protection of personal data and privacy. At the time of protocol submission, child and caregiver endline assessments were underway but not complete and no data had been analysed.

## Supplementary Information

Supplementary Material 1. SPIRIT 2025 checklist of items.

Supplementary Material 2. List of health centres included in the trial and the total populations served.

## Data Availability

Anonymised data will be made available on the London School of Hygiene & Tropical Medicine Data Compass (datacompass.lshtm.ac.uk). Data will be made available upon request, provided the requester has a legitimate purpose for using the data. Availability of data will be contingent on relevant approvals under Rwanda Law No 058/2021 relating to the protection of personal data and privacy.

## Abbreviations

AE: Adverse events
cRCT: Cluster randomised controlled trial
DAZ: Development-for-age Z-score
DMC: Data Monitoring Committee
GDPR: General Data Protection Regulation
GMCD: Guide for Monitoring Child Development
GSED: Global Scales for Early Development
ICC: Intra-cluster correlation
LMIC: Low- and middle-income country
MDAT: Malawi Development Assessment Tool
MICS: Multiple Indicator Cluster Survey
OFC: Occipital Frontal Circumference
OPD: Organisation of persons with disabilities
PDC: Pediatric Development Clinic
PEDI-CAT: Pediatric Evaluation of Disability Inventory—Computer Adaptive Test
PedsQL: Pediatric Quality of Life Inventory
PIH/IMB: Partners in Health/Inshuti mu Buzima
SAE: Serious adverse events
SD: Standard deviation
SRQ: Self-Reporting Questionnaire
TSC: Trial Steering Committee
YC-PEM: Young Children’s Participation and Environment Measure
