# Supplementary material for: Early childhood intervention for children at risk of developmental disabilities and their caregivers in Rwanda: study protocol for the PDC/Baby Ubuntu cluster randomised trial
Source: Trials. 2026 Mar 20;27:240. doi: 10.1186/s13063-026-09613-7 (PMC13023207; doi:10.1186/s13063-026-09613-7)
Supplement: Supplementary file 2 — Supplementary Material 2. List of health centres included in the trial and the total populations served. [file 13063_2026_9613_MOESM2_ESM.pdf]

Additional File 2: List of health centres included in the trial and the total populations served

Burera District		Kamonyi District		Gakenke District	
Health Centre	Population	Health Centre	Population	Health Centre	Population
Bungwe	16,322	Cyeru	17,899	Busengo	22,610
Butaro	32,082	Gihara	72,778	Bushoka	21,318
Cyanika	39,739	Kabuga	5,910	Coko	7,564
Gahunga	28,059	Kamonyi	36,859	Cyabingo	19,867
Gatebe	19,544	Karama	20,879	Gatonde	20,422
Gitare	23,089	Karangara	10,506	Janja	8,679
Kinoni	6,533	Kayenzi	25,209	Kamubuga	24,101
Kinyababa	23,746	Kayumbu	17,106	Karambo	14,225
Kirambo	19,435	Kigese	59,952	Mataba	17,225
Kivuye	7,577	Mugina	27,995	Minazi	14,519
Mucaca	20,920	Musambira	42,198	Muhondo	21,825
Ndongozi	14,719	Nyagihamba	28,225	Muyongwe	9,547
Ntaruka	12,517	Nyamiyaga	45,645	Nemba	17,101
Nyamugali	21,401	Remera Rukoma	39,688	Nganzo	26,207
Rugarama	27,051	**Total Population**	**450,849**	Nyange	10,791
Ruhombo	11,531			Nyundo	19,685
Ruhunde	20,157			Rukura	9,599
Rusasa	9,278			Ruli	22,981
Rwerere	21,611			Rushashi	12,107
**Total Population**	**375,311**			Rusoro	22,318
				Rutake	7,696
				Rutenderi	19,877
				Rwankuba	8,922
				**Total Population**	**379,186**
